# Cabazitaxel suppresses colorectal cancer cell growth via enhancing the p53 antitumor pathway

**DOI:** 10.1002/2211-5463.13290

**Published:** 2021-10-11

**Authors:** Wen Zhang, Ruiqian Sun, Yongjun Zhang, Rong Hu, Qian Li, Weili Wu, Xinyu Cao, Jiajian Zhou, Jianfeng Pei, Ping Yuan

**Affiliations:** ^1^ Guangdong Provincial Key Laboratory of Colorectal and Pelvic Floor Disease The Sixth Affiliated Hospital of Sun Yat‐sen University Guangzhou China; ^2^ Guangdong Institute of Gastroenterology Guangzhou China; ^3^ Guangdong Country Garden School Foshan City China; ^4^ Dermatology Hospital Southern Medical University Guangzhou China; ^5^ Institute of Clinical Medical Sciences,， Center of Respiratory Medicine China‐Japan Friendship Hospital Beijing China; ^6^ Center for Quantitative Biology,， Academy for Advanced Interdisciplinary Studies Peking University Beijing China

**Keywords:** cabazitaxel, colorectal cancer cell, HCT116, RNA‐sequencing, xenograft

## Abstract

There were approximately 1.93 million new cases and 940 000 deaths from colorectal cancer in 2020. The first‐line chemotherapeutic drugs for colorectal cancer are mainly based on 5‐fluorouracil, although the use of these drugs is limited by the development of drug resistance. Consequently, there is a need for novel chemotherapeutic drugs for the efficient treatment of colorectal cancer patients. In the present study, we screened 160 drugs approved by the Food and Drug Administration and identified that cabazitaxel (CBT), a microtube inhibitor, can suppress colony formation and cell migration of colorectal cancer cells *in vitro*. CBT also induces G2/M phase arrest and apoptosis of colorectal cancer cells. Most importantly, it inhibits the growth of colorectal cancer cell xenograft tumors *in vivo*. Transcriptome analysis by RNA‐sequencing revealed that Tub family genes are abnormally expressed in CBT‐treated colorectal cancer cells. The expression of several p53 downstream genes that are associated with cell cycle arrest, apoptosis, and inhibition of angiogenesis and metastasis is induced by CBT in colorectal cancer cells. Overall, our results suggests that CBT suppresses colorectal cancer by upregulating the p53 pathway, and thus CBT may have potential as an alternative chemotherapeutic drug for colorectal cancer.

## Graphical abstract

In the present study, we screened 160 drugs approved by the Food and Drug Administration, aiming to identify novel chemotherapeutic agents for colorectal cancer (CRC) treatment. We identified that the microtubule inhibitor cabazitaxel (CBT) has a suppressive effect on CRC cells both *in vitro* and *in vivo*. Transcriptome analysis revealed that CBT induced apoptosis in CRC cells by upregulating the p53 pathway, implying that CBT may hold chemotherapeutic potential for the treatment of patients with CRC.

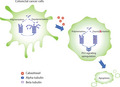

**Abbreviations**
5‐FU: 5‐fluorouracil
CBT: cabazitaxel
CI: confidence interval
CRC: colorectal cancer cell
GFP: green fluorescent protein
GO: Gene Ontology
GSEA: geneset enrichment analysis
IC_50_
: half‐maximal inhibitory concentration
KEGG: Kyoto Encyclopedia of Genes and Genomes
KO: knockout
MTT: 3‐(4,5‐dimethylthiazol‐2‐yl)‐2,5‐diphenyl‐tetrazolium bromide
RNA‐seq: RNA‐sequencing

Colorectal cancer is the third most diagnosed cancer and leads to the second greatest mortality among cancers worldwide. There were approximately 1.93 million new cases and 940 000 deaths of colorectal cancer in 2020 according to the World Health Organization 1. Multiform therapeutic strategies, such as surgery, chemotherapy, radiotherapy and recent immunotherapy, have been developed and applied to colorectal cancer patients. Surgical resection plus chemotherapy is the most common treatment for early stage of primary colorectal cancer [2]. The first‐line chemotherapeutic drugs of colorectal cancer are mainly based on 5‐fluorouracil (5‐FU). However, these drugs exhibit compromised efficacy as a result of significant toxicity, drug resistance or patient inconvenience [3]. The high mortality of colorectal cancer indicates that the current therapy is far from ideal. Novel chemotherapeutic drugs for the efficient treatment of colorectal cancer patients are urgently needed.

As the safety of Food and Drug Administration (FDA)‐approved drugs is demonstrated, the exploration of their therapeutic application to colorectal cancer can greatly reduce the cost and time for drug application. Cabazitaxel (CAS183133‐96‐2; RPR 116258; XRP6258; TXD258; Jevtana; CBT) is an FDA‐approved drug for the treatment of patients who are diagnosed with metastatic castration‐resistant prostate cancer that is resistant to paclitaxel and docetaxel treatment [4]. CBT is a semi‐synthetic taxane derivative. It promotes the polymerization of tubulin and stabilizes microtubules. It inhibits prostate cancer cells by inhibiting androgen receptor and heat shock protein [5] and shows antitumor activity in docetaxel‐refractory metastatic prostate cancer and breast cancer [6, 7]. It is also reported to induce autophagy via the phosphoinositide 3‐kinase/Akt/mechanistic target of rapamycin pathway in lung adenocarcinoma cancer cell line A549 [8]. However, it is not clear whether CBT is effective in inhibiting colorectal cancer, nor what the underlying mechanism comprises.

HCT116 cell is a commonly used colorectal cancer cell line. It can be cultured without growth factors *in vitro* [9, 10, 11, 12]. HCT116 cells are highly motile and invasive and showed high efficiency with respect to forming tumors in a subcutaneous xenograft experiment [13]. Using this cell line, we screened 160 FDA‐approved drugs and found that CBT can efficiently inhibit HCT116 cells. Employing a series of *in vitro* assays, we found that CBT can suppress HCT116 cell proliferation and migration. CBT induces G2/M phase cell cycle arrest and apoptosis of HCT116 cells. Most interestingly, CBT can efficiently inhibit tumor growth in the HCT116 cell xenograft mouse model. By comparing the transcriptome of CBT‐treated and control HCT116 cells, we found that CBT treatment leads to upregulation of genes involved in the p53 signaling pathway. Further knockout (KO) of p53 in HCT116 cells confirms the key role of p53 signaling for the CBT inhibitory effect in colorectal cancer cells.

Overall, the present study reports a novel anti‐colorectal cancer role for CBT, which may be used as an alternative chemotherapeutic drug for the efficient treatment of colorectal cancer patients.

## Materials and methods

### Cell culture

HCT116 cells were cultured in Dulbecco’s modified Eagle's medium basic media supplemented with 10% fetal bovine serum at 37 °C in an incubator with 5% CO_2_. The cells were passaged by 0.25% trypsin at a ratio of 1:3 after confluency.

### Drug screening by the 3‐(4,5‐dimethylthiazol‐2‐yl)‐2,5‐diphenyl‐tetrazolium bromide (MTT) cytotoxicity assay

5 × 10^3^ HCT116 colorectal cancer cells were seeded per well in a 96‐well plate for overnight culture to allow the cells to adhere to the plate. Dimethylsulfoxide or dimethylsulfoxide diluted FDA drugs were added to the culture medium separately on the next day. After 48 h, cell viability was assessed by the MTT colorimetric assay. Ten microliters of MTT solution (5 mg·mL^−1^ in PBS) were added to each well. After 3 h of incubation, absorbance at 570 nm with a reference wavelength of 690 nm was assessed to calculate the cell viability with respect to the untreated cells. Three independent biological experiments were performed for each assay.

### Cabazitaxel solution preparation

Cabazitaxel was purchased from Topscience (Shanghai, China). For the *in vitro* experiment, 5 mg of CBT was dissolved in 0.598 mL of dimethylsulfoxide (10 mm) and further diluted with PBS to different concentrations. For the *in vivo* experiment, based on the formulation of Jevtana (Sanofi‐Aventis Groupe, Paris, France), 10 mg of CBT was dissolved in 0.26 g of polysorbate 80 (Tween 80) and mixed with 0.95 mL of 13% ethanol (w/w) in ddH_2_O before injection, wiith 0.9% sodium chloride solution being used in the final dilution.

### Half‐maximal inhibitory concentration (IC_50_) measurement

The indicated colorectal cancer cells and prostate cancer cells were treated with a series of diluted CBT for 48 h. The cell viability was measured by the MTT colorimetric assay. The values of CBT‐treated samples were normalized with untreated samples in excel (Microsoft Corp., Redmond, WA, USA) and then input in a nonlinear sigmoidal curve of prism, version 7 (GraphPad Software Inc., San Diego, CA, USA) to calculate the IC_50_. Three independent biological experiments were performed for each assay.

### RNA extraction and RT cDNA synthesis

Total RNA was extracted from the control or CBT‐treated cells using TRIzol Reagent (Invitrogen, Waltham, MA, USA). The concentration and purity of RNA was measured by spectrophotometry (Nanodrop Technologies, Inc., Wilmington, DE, USA). cDNA was synthesized from 2 µg of RNA using 5 × PrimeScript RT Master Mix (Takara, Shiga, Japan) in accordance with the manufacturer’s instructions.

### Western blotting

Total proteins of the cells were harvested with RIPA buffer and separated via SDS/PAGE. Subsequently, the proteins were transferred to a poly(vinylidene difluoride) membrane and blocked by 5% slim milk in TBS plus 0.1% Tween 20. The membrane was then blot with β‐actin antibody (dilution 1:1000; SC47778; Santa Cruz Biotechnology, Santa Cruz, CA, USA), Phospho‐Histone H2A.X antibody (dilution 1:1000; catalogue no. 2577; Cell Signaling Technology, Danvers, MA, USA) or p21/Waf1/Cip1 antibody (dilution 1:1000; catalogue no. 2947; Cell Signaling Technology), respectively, overnight at 4 °C. The membrane was washed with TBS plus 0.1% Tween‐20 solution and then blotted with proper horseradish peroxidase‐conjugated secondary antibodies. After washing, chemdoc (Bio‐Rad, Hercules, CA, USA) was used to detect the signals.

### Real‐time PCR

The relative expression of mRNA was examined by real‐time PCR using SYBR Green Master Mix (Takara) on an ABI QuantStudio™ 7 real‐time PCR system (Thermo Fisher Scientific, Wlathm, MA, USA). The thermal cycling conditions included an initial hold period at 95 °C for 30 s followed by a two‐step PCR program, comprising 95 °C for 5 s and 60 °C for 30 s with 40 cycle repeats. To evaluate the relative expression, the Ct value of the examined sample gene was first normalized with the Ct value of endogenous Gapdh and then with the Ct values of the respective control sample gene. All experiments were performed with three biological repeats and three technique repeats. Student's *t*‐test was used for statistical analysis. The primer sequences for real‐time PCR are provided in Table 1.

**Table 1:** Sequences of quantitative RT‐PCR primers and TP53 gRNA oligos.

RT‐PCR primer			
Gene	Species	Forward	Reverse
*Tp53i3*	Human	AATGCTTTCACGGAGCAAATTC	TTCGGTCACTGGGTAGATTCT
*Gadd45a*	Human	CCCTGATCCAGGCGTTTTG	GATCCATGTAGCGACTTTCCC
*Pmaip1*	Human	ACCAAGCCGGATTTGCGATT	ACTTGCACTTGTTCCTCGTGG
*Cdkn1a*	Human	TGTCCGTCAGAACCCATGC	AAAGTCGAAGTTCCATCGCTC
*Fas*	Human	AGATTGTGTGATGAAGGACATGG	TGTTGCTGGTGAGTGTGCATT

TP53 gRNA oligo		
Name	Species	Sequence
gRNA1	Human	CCATTGTTCAATATCGTCCG
gRNA2	Human	CCATTGCTTGGGACGGCAAG

### Apoptosis assay

The apoptosis assay was performed using an Annexin V‐FITC/PI Apoptosis Kit (MultiSciences Biotech, Hangzhou, China). Cancer cells were seeded at 1 × 10^5^ cells per well in a six‐well plate for overnight culture. Then, the cells were treated with PBS and cabazitaxel at the IC_50_ of the respective cells for 48 h. Next, cells were collected for the apoptosis assay in accordance with the manufacturer’s instructions. Flow cytometry analysis was performed using a FACSCanto II (BD Biosciences, Franklin Lakes, NJ, USA) flow cytometer. The percentage of cells at different cell cycle phases was analyzed using flowjo (https://www.flowjo.com). Three biological repeats were employed for each experiment. Student's *t*‐test was used for the statistical analysis.

### Cell cycle assay

Cancer cells were seeded at 1 × 10^5^ cells per well in a six‐well plate. After overnight culture, the cells were treated with cabazitaxel at the IC_50_ of the respective cell line for 48 h. The cells were gently lifted with 0.25% Trypsin (Gibco, Waltham, MA, USA) at 37 °C for 1 min. Next, the cells were washed once with PBS and fixed with 75% alcohol at −20 °C overnight. Subsequently, the cells were centrifuged at 395 ***g*** for 5 min and suspended in propidium iodide solution (50 µg·mL^−1^ propidium iodide, 0.1 mg·mL^−1^ RNase A and 0.05% Triton X‐100 in PBS) and incubated at 37 °C for 40 min. After centrifugation, the supernatant was removed and the cells were resuspended in 500 µL of PBS for flow cytometry analysis using a FACSCanto II (BD Biosciences) flow cytometer. The percentage of cells at different cell cycle phases was analyzed using flowjo. Three biological repeats were tested for each experiment. Student’s *t*‐test was used for statistical analysis.

### Colony formation assay

Agarose mixture containing 0.5 mL of growth media plus 20% fetal bovine serum and 0.5 mL of 0.8% agararose gel was used to coat each well of a six‐well plate. The plates were subsequently cooled at 4 °C for 5 min to solidify the agarose and then transferred to the tissue culture hood and warmed to 37 °C. 5 × 10^3^ HCT116 cells thoroughly mixed with low density agarose mixture containing 0.5 mL of growth media plus 20% fetal bovine serum and 0.5 mL of 0.4% agararose gel were added to each well of the agarose‐coated plates. After solidification for another 20 min, complete media (1 mL) plus cabazitaxel at different concentrations was added to the wells. After 72 h, the media containing cabazitaxel was removed and 1 mL of fresh media was used for replenishment. The medium was changed every 3 days up to day 14. Colonies were stained with 0.05% crystal violet for 1 h and then washed with PBS. The images of the colonies were captured using a microscope (Olympus, Tokyo, Japan) with a 4× objective lens. The number of colonies was counted manually. The area of colonies was quantified using imagej (NIH, Bethesda, MD, USA). The experiments were performed with three biological repeats and three technical repeats.

### Wound healing assay

3 × 10^4^ HCT116 cells were seeded in each well in 12‐well plates with Culture‐Insert 4 Well silicone inserts (Ibidi, Gräfelfing, Germany). The cells were incubated at 37 °C and 5% CO_2_ for 24 h for attachment. The Culture‐Insert 4 Well was then removed with sterile tweezers. Growth medium with or without 0.03 μm cabazitaxel was added to the culture. All experiments included three biological repeats. The culture images were captured at different time points using a microscope (Leica, Wetzlar, Germany). The gap of the culture was measured using imagej.

### *In* *vivo* antitumor assay

All animal experiments were approved by the Institutional Animal Care and Use Committee of the Sixth Affiliated Hospital of Sun Yat‐sen University (Guangzhou, China). Five‐week‐old female BALB/c nude mice were purchased from Charles River Laboratories (Beijing, China) and maintained under specific pathogen‐free condition under a 12:12‐h dark/light photocycle. A maximum of five mice were kept in one microisolator cage with *ad libitum* feeding of autoclaved food and water. One hundred microliters of green fluorescent protein (GFP)‐labeled HCT116 cells in PBS at a concentration of 5 × 10^4^ cells·μL^−1^ were subcutaneously injected into the left flank of 6‐week female mice anesthetized using inhaled isoflurane. Seven days later, the xenografted tumors grew to approximately 30–200 mm^3^ in size. The mice were randomly assigned to five groups (*n* = 3 per group) for the administration of different reagents. Intraperitoneal injections with 8 and 16 mg·kg^−1^ CBT, 8 and 16 mg·kg^−1^ 5‐FU and PBS were performed, respectively, at days 0, 5 and 10 after group assignment. The growth of tumor was monitored with an *in vivo* imaging system (IVIS Spectrum; Xenogen, Alameda, CA, USA) after the mice were anesthetized using inhaled isoflurane. Tumor volume was measured every 3 days and calculated as *V* = (length × width × height)/2. The mice were weighed every 3 days and their general physical status was recorded daily. The experiment was terminated before the tumor size reached 2000 mm^3^. The mice were killed with CO_2_ and the tumors were dissected out for the subsequent experiments.

### Gene expression analysis

RNA was extracted from the indicated cells. The RNA‐sequencing (RNA‐seq) libraries were constructed and sequenced with NovaSeq 6000 sequencer by Berry Genomics Co Ltd (Beijing, China). Raw sequencing reads were subjected to quality filtering and adapter removal. The remain reads were then aligned to the reference human genome (hg19) using star2 (v2.7.3a) [14]. The gene expression was quantified as FPKM (i.e. fragments per kilobase of gene per million mapped read) using cufflinks, version 2.2.1 [15]. Differential expression genes were determined using |log2(fold change)| ≥ 0.58 in CBT‐treated HCT116 cells versus control HCT116 cells. The |log2(fold change)| prerank gene list was used for the subsequent enrichment analyses. Geneset enrichment analysis (GSEA) was used to assess the enrichment from the Hallmark geneset collection provided by the v4.0 MsigDB [16] and Kyoto Encyclopedia of Genes and Genomes (KEGG) [17, 18, 19].

### Establishment of TP53 KO HCT116 cells by CRISPR/Cas9

TP53 KO HCT116 cells were generated by CRISPR/Cas9 using gRNAs as described previously [20]. Two hTP53 gRNA KO plasmids (YKO‐RP003‐hTP53, YKO‐RP003‐hTP53, Ubigene) were obtained from Ubigene Company (Guangzhou, China). gRNA oligos are listed in Table 1. The hTP53 gRNA KO plasmids were transfected into HCT116 cells using Lipofectamine 3000 (Invitrogen). The cells were selected using a concentration of 0.8 μg·mL^−1^ of purimycin at 24 h after transfection to eliminate the nontransfected cells. The survived cells were subcultured and checked for expression of GFP. Knockout of TP53 was confirmed by western blotting.

## Results

### Cabazitaxel can efficiently inhibit the proliferation and migration of colorectal cancer cells

**Fig. 1:**
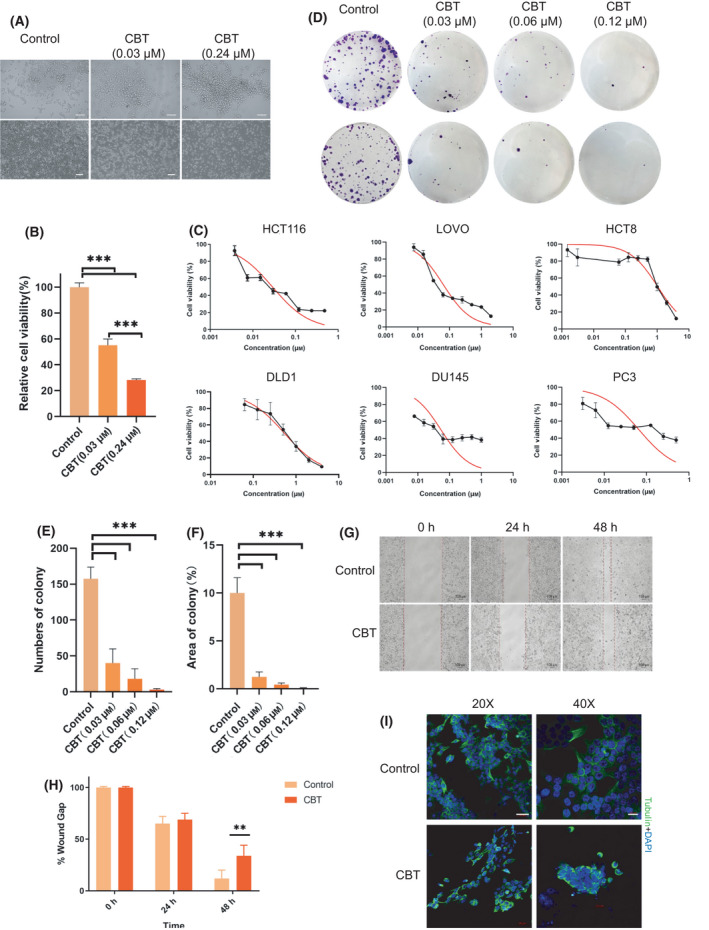
CBT can efficiently inhibit colorectal cancer cells. (A) Cell morphology of control and 0.03 and 0.24 μm CBT‐treated HCT116 cells at high magnification (Top: scale bar = 100 μm) and log magnification (Bottom: scale bar = 150 μm). (B) Relative cell viability measured by the MTT assay. Error bars indicate the SD. Student's *t*‐test was used for statistical analysis. Data are mean ± SD (*n* = 6). ***P < 0.001. (C) *In vitro* cytotoxicity of CBT at a different concentration to human colorectal cancer cells HCT116, LOVO, HCT8 and DLD1, as well as Du145 and PC3 prostate cancer cells. Data are the mean ± SD(*n* = 6). Red lines indicate the nonlinear fit sigmoidal curve. The cell viability rate was obtained by normalizing the MTT assay output of CBT‐treated cells with corresponding dimethylsulfoxide‐treated cells. (D) Colony morphology of control HCT116 cells and CBT (at the indicated concentration)‐treated HCT116 cells. (E) The number of colonies formed by HCT116 cells after treatment with control or CBT of the indicated concentration. Error bars indicate the SD. Student's *t*‐test was used for statistical analysis. Data are the mean ± SD (*n* = 3). ***P < 0.001. (F) Percentage of control or CBT‐treated HCT116 cell formed colony area in the total cell culture plate area. The area was measured using imagej. Error bars indicate the SD. Student's *t*‐test was used for statistical analysis. Data are the mean ± SD (*n* = 3). ***P < 0.001. (G) Microscopic images of the wound‐healing assay with control HCT116 cell culture and 0.03 μm CBT‐treated cell culture at the indicated time. Scale bar = 200 μm. (H) Quantification of wound gap in control HCT116 cell culture and 0.03 μm CBT‐treated cell culture at different time points compared to the wound gap at 0 h. Data are the mean ± SD (*n* = 3). **P < 0.01. (I) Representative immunofluorescence images of control and CBT‐treated cells blotted with antibody against tubulin. Nuclear DNA was counterstained with DAPI. Scale bar in the 20‐fold magnified image = 50 μm, whereas the scale bar in the 40‐fold magnified image = 20 μm.

To identify drugs that have potential to treat colorectal cancer, we utilized HCT116 cells as a colorectal cancer cell model and screened 160 FDA‐approved drugs (Table 2). Our initial trial revealed that CBT could efficiently reduce the number of viable HCT116 cells after 48 h of drug treatment (Fig. 1A). A concentration of 0.03 μm CBT reduced the viable HCT116 cells to 50%, whereas 0.24 μm CBT reduced the cell viability to 30% (Fig. 1B). To determine whether CBT plays a broad inhibitory role for different colorectal cancer cells, we next investigated its cytotoxicity to HCT116, LoVo, HCT8 and DLD1 cells. Because CBT is an FDA‐approved drug for prostate cancer, we also included prostate cancer cell DU145 and PC3 in the experiment as positive controls. We examined cell viability at 48 h after CBT treatment at different concentrations by the MTT colorimetric assay and calculated the IC_50_. IC_50_ values of CBT to HCT116, LoVo, HCT8 and DLD1cells were 0.029 μm [0.023–0.036 μm, 95% confidence interval (CI)], 0.063 μm (0.047–0.087 μm, 95% CI), 0.255 μm (0.198–0.328 μm, 95% CI) and 0.532 μm (0.438–0.646 μm, 95% CI), respectively. Meanwhile, IC_50_ values of CBT to prostate cancer cell DU145 and PC3 cells were 0.054 μm (0.033–0.090 μm, 95% CI) and 0.066 μm (0.030–0.148 μm, 95% CI) (Fig. 1C). These results suggest that CBT inhibits colorectal cancer cell HCT116 and LoVo cells as efficiently as prostate cancer cell DU145 and PC3 cells. However, a much higher dose of CBT is required to inhibit colorectal cancer cell HCT8 cells and DLD1 cells.

**Table 2:** FDA‐approved drugs for colorectal cancer screen.

Drug name									
Nitisinone	Methacholine chloride	Dutasteride	Edoxaban tosylate monohydrate	Ipragliflozin	Vemurafenib (PLX4032, RG7204)	AP24534 Ponatinib	Montelukast sodium	Panobinostat (LBH589)	Edoxaban
Pomalidomide	Ciclesonide (RPR251526)	WY‐14643 (Pirinixic Acid)	Isavuconazole	Brexpiprazole	Trelagliptin succinate	Afatinib (BIBW2992)	Indacaterol	Felbamate	MLN2238 (Ixazomib)
Nandrolone	Flupenthixol dihydrochloride	Guanethidine monosulfate	Nandrolone decanoate	TAK‐438 (Vonoprazan fumarate)	Diflorasone	Etonogestrel	Ulipristal acetate	Etravirine (TMC125)	Etofibrate
Dexmedetomidine	Ivabradine hydrochloride	Dienogest	Rufinamide	Ecabet sodium	Oxandrolone	Dalasetron mesylate hydrate	Peramivir trihydrate	Bazedoxifene acetate	Eflornithine hydrochloride monohydrate
Lercanidipine	Fluoxymesterone	Lacosamide	Prucalopride	Pitavastatin calcium	Tirofiban hydrochloride monohydrate	Allylestrenol	Alosetron hydrochloride	Aclidinium bromide	Tolmetin
Alizapride hydrochloride	Tapentadol hydrochloride	Cabazitaxel	Alcaftadine	Levosimendan	Pazopanib	Amfenac sodium monohydrate	Entecavir	Neomycin sulfate B	Pimobendan
Carbidopa hydrate	Benactyzine hydrochloride	Levomilnacipran	Nafamostat mesylate	Ziprasidone	Fasudil (HA‐1077) hydrochloride	Daclatasvir dihydrochloride	Deoxycorticosterone acetate	Fimasartan (BR‐A‐657)	Menatetrenone
Avanafil	Acotiamide hydrochloride	Ebastine	Tolvaptan	Elvitegravir (GS‐9137, JTK‐303)	Efinaconazole	Radotinib	Dolutegravir sodium (GSK1349572)	Rilpivirine	Amikacin sulfate salt
Nicotinic acid hydrazide	Hesperetin	Drofenine hydrochloride	Luteolin	Formononetin	Isoliquiritigenin	Fulvestrant	Tranilast	Oxfendazole	Gallamine triethiodide
Lisinopril dihydrate	Cyclophosphamide monohydrate	Pimaricin	l‐Cycloserine	Naftopidil	Ursodeoxycholic acid	Broxiquinoline	Gabapentin	Cyanoacetohydrazide	Simvastatin
Ganciclovir	d‐Phenylalanine	Quinine	Allopurinol	Niflumic acid	Pranlukast	Ketoconazole	Lamotrigine	Rifampicin	Mevastatin
Ribavirin	Orlistat	Hydroquinone	Hydroxyurea	Chloral hydrate	Tilmicosin	Phenothiazine	Coumarin	Aptal	Troxerutin
Casanthranol	Carbadox	Piroxicam	Omeprazole	Salicylanilide	Sulfisoxazole	Sulfameter	Orotic acid	dl‐Carnitine	Ethosuximide
Pyrithioxin	Pindolol	l‐Ornithine	Urea	Dimetridazole	Acetylcysteine	Gallic acid	Diosmin	Nicotinic acid	d‐Camphor
Behenic alcohol	Sulfamethoxazole	d(+)‐Glucose	Mefenamic acid	Tinidazole	Thiamine hydrochloride	Oxytetracycline (Terramycin)	Furaltadone hydrochloride	Chloroxylenol	Ofloxacin
Adrenosterone	Cepharanthine	Guaifenesin	Geniposide	l‐5‐Hydroxytryptophan	Doxycycline hyclate	Salicylic acid	Batyl alcohol	Magnolol	Gastrodin

Next, we examined the effect on colony formation. The number of HCT116 colonies decreased gradually with an increase in CBT concentration (Fig. 1D–F). To investigate whether CBT can inhibit the migration of colorectal cancer cells, we then examined the effect of CBT on cell motility by the wound‐healing assay using HCT116 cells. An Ibidi culture insert was used to generate the wound gap and serum‐free culture medium was added to the cells after the insert was removed to reduce the effect of cell proliferation. Obviously, the Ibidi culture insert generated gap demonstrated much slower closing for CBT pretreated colorectal cancer cells than for control cells at 48 h after insert removal (Fig. 1G,H). Immunostaining of tubulin revealed that CBT‐treated HCT116 cells showed cytoskeleton disorder and morphological malformation with a reduced pseudopod, which is line with the reduced motility of CBT‐treated cells. (Fig. 1I). Taken together, CBT can efficiently inhibit the growth and migration of colorectal cancer cells.

### CBT induces G2/M phase cell cycle arrest and apoptosis in colorectal cancer cells

**Fig. 2:**
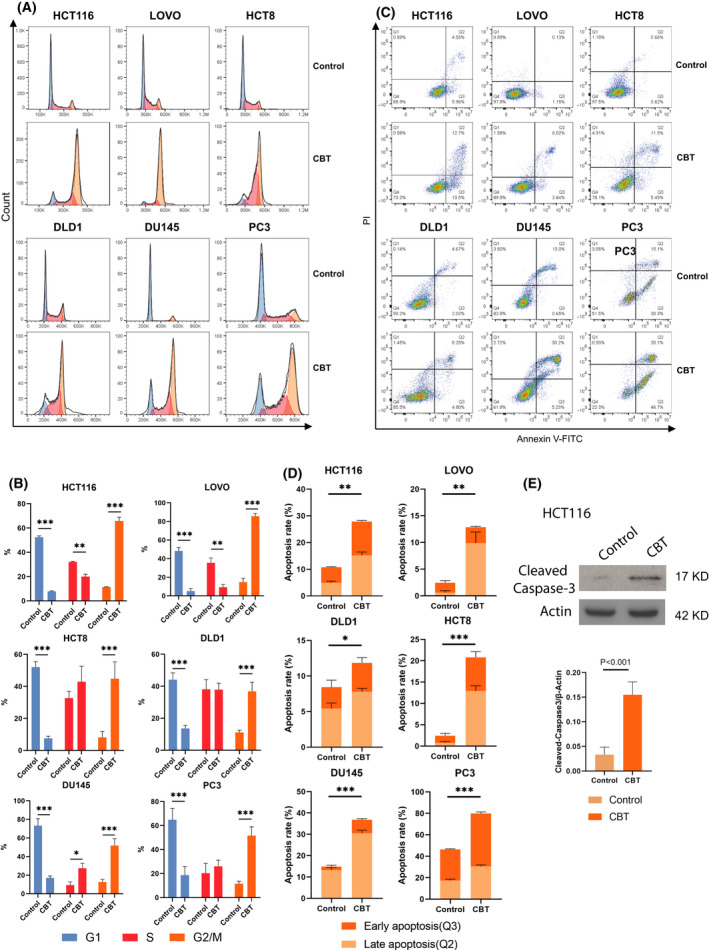
CBT induced G2/M arrest and apoptosis in colorectal cancer cells. (A) Cell cycle distributions of control and CBT‐treated HCT116, LOVO, HCT8 and DLD1 human colorectal cancer cells, as well as DU145 and PC3 prostate cancer cells, by flow cytometry analysis. Blue represents G1 phase; red represents S phase and orange represents G2/M phase. (B) Percentage of cells at G1 phase, S phase and G2/M phase in (A). Data are the mean ± SD (*n* = 3). *P < 0.05, **P < 0.01, ***P < 0.001. (C) Representative graphs of cell apoptosis of control and CBT‐treated HCT116, LOVO, HCT8 and DLD1 human colorectal cancer cells, as well as DU145 and PC3 prostate cancer cells, examined by double staining with propidium iodide and annexin V‐fluorescein isothiocyanate and a flow cytometry assay. (D) Early and late apoptosis rate of (C). Student's *t*‐test was used for statistical analysis Data are the mean ± SD (*n* = 3). *P < 0.05, **P < 0.01, ***P < 0.001. (E) Representative image of western blotting (left) and densitometric analyses (right) of the expression of cleaved caspase‐3 expression in CBT‐treated and nontreated HCT116 cells. Actin was used as an internal control. Data are the mean ± SD (*n* = 2).

To determine how CBT suppresses colorectal cancer cell proliferation, we a performed flow cytometry assay to examine the effect of CBT on the cell cycle distribution of colorectal cancer cells and prostate cancer cells. The CBT concentration at IC_50_ to the respective cell lines was adopted for the assay. As expected, CBT treatment led to G2/M cell cycle arrest in all tested cell lines (Fig. 2A). There were approximately 3‐fold more cells at G2/M phase in CBT‐treated cells than in the control cells (Fig. 2B). This observation is consistent with previous studies reporting that CBT causes G2/M cell cycle arrest in cancer cells [21, 22]

Because CBT induced G2/M phase arrest, we next investigated whether CBT treatment triggers apoptosis of colorectal cancer cells. Similarly, the CBT concentration at IC_50_ to the respective cell lines was adopted for the assay. Forty‐eight hours after CBT treatment, the control and CBT‐treated cells were stained with annexin V‐fluorescein isothiocyanate and propidium iodide to analyze the apoptosis rate of these cells via flow cytometry. Compared to the control cells, CBT treatment led to an approximately 2‐fold or more increase in cell apoptosis (Fig. 2C,D). Western blotting revealed that the apoptosis marker‐cleaved caspase‐3 was also greatly increased in CBT‐treated HCT116 cells (Fig. 2E).

### Cabazitaxel inhibits tumor growth in colorectal cancer xenograft model

**Fig. 3:**
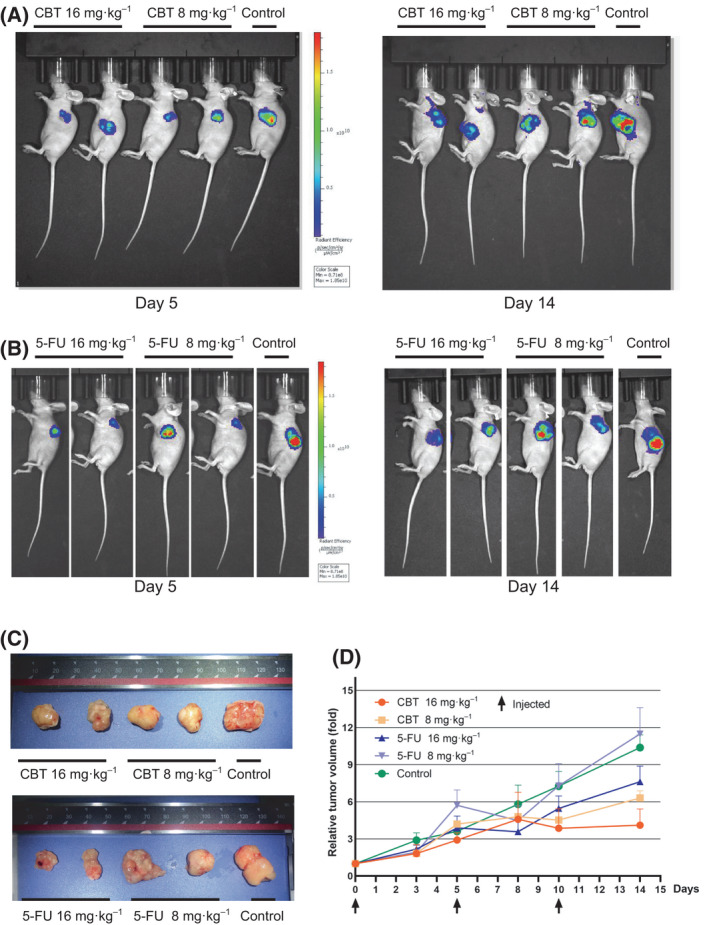
Cabazitaxel inhibits tumor growth in a colorectal cancer xenograft model. (A) Representative AVIS images showing control, 8 and 16 mg·kg^−1^ CBT‐treated mice that bear HCT116 xenograft tumor at days 5 and 14 after the first injection. (B) Representative AVIS images showing control, 8 and 16 mg·kg^−1^ 5‐FU treated mice that bear HCT116 xenograft tumor at days 5 and 14 after the first injection. (C) Representative pictures of tumors harvested from CBT, 5‐FU and control treated mice. (D) Relative tumor growth fold of CBT, 5‐FU and control treated mice. The relative tumor growth fold was obtained by normalizing the tumor volume at each time point with the tumor volume at the injection starting time. Data are the mean ± SD (*n* = 3).

To evaluate the antitumor effect of cabazitaxel against colorectal cancer, we subcutaneously injected GFP‐labeled HCT116 cells into nude mice to derive a xenograft model of colorectal cancer. Based on the dose of Jevtana (cabazitaxel) used for the patient, the dose of CBT utilized for the mouse experiments was derived according to the body surface area [23, 24, 25]. Accordingly, 8 and 16 mg·kg^−1^ CBT were tested for the efficacy. Because 10–40 mg·kg^−1^ 5‐FU was reported to be effective in inhibiting tumor growth [26, 27], we utilized 8 mg·kg^−1^ 5‐FU as a negative drug control and 16 mg·kg^−1^ 5‐FU as a positive drug control for the assay. Mice injected with PBS were also used as a negative control. All experimental mice had xenograft tumors at day 7 after subcutaneous injection of HCT116 cells. We then randomized the mice and treated them with CBT and 5‐FU, respectively. Using IVIS Spectrum to monitor tumor growth, we found that mice treated with 8 mg·kg^−1^ CBT, 16 mg·kg^−1^ CBT and 16 mg·kg^−1^ 5‐FU showed relatively smaller xenograft tumors than those treated with 8 mg·kg^−1^ 5‐FU and PBS (i.e. negative control groups) (Fig. 3A,B). After further analyses of the drug efficacy by normalizing the tumor with tumor at the injection starting point, we concluded that 8 and 16 mg·kg^−1^ CBT can inhibit HCT116 cell formed tumors in nude mice as efficiently as 16 mg·kg^−1^ 5‐FU (Fig. 3C,D).

### Cabazitaxel treatment induces the abnormal expression of Tubb family gene expression in colorectal cancer cells

**Fig. 4:**
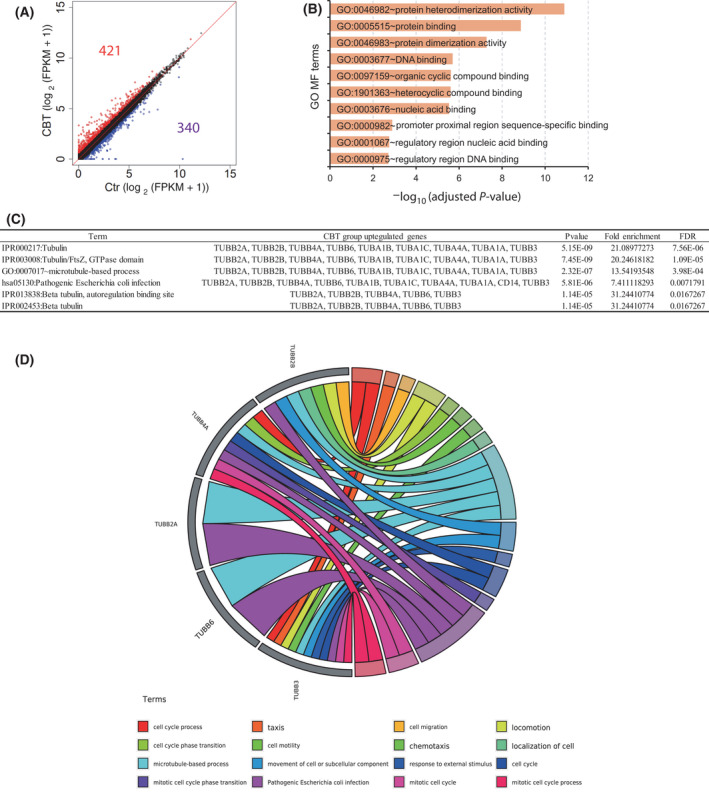
Cabazitaxel inhibits colorectal cancer cell growth via activating the P53 signaling pathway. (A) Scatter plot showing the upregulated genes (red points) and downregulated genes (blue points) in CBT‐treated HCT116 cells compared to control cells. The cut‐off for expression difference is 0.58. (B) GO enrichment analysis showing the enriched molecular of upregulated genes in CBT‐treated HCT 116 cells compared to control HCT116 cells. GO terms of upregulated and downregulated differentially expressed genes were assessed separately for enrichment using Fisher's exact test. (C) DAVID analysis of the enriched TUBB and TUBA family related genes. (D) Function chord plot of CBT induced upregulated TUBB family genes.

To further investigate why CBT can efficiently inhibit colorectal cancer, we performed RNA‐seq assays to determine the transcriptomic changes between the control and CBT‐treated HCT116 cells. Compared to the control, 421 genes were upregulated and 340 genes were downregulated in CBT‐treated HCT116 cells (Fig. 4A and Table 3).

**Table 3:** Differential expressed genes in CBT‐treated HCT116 cells.

Gene name								
CBT vs Control upregulated genes								
CDC37L1‐AS1	GADD45B	CDC20	CEACAM1	ACTA2	MIR4435‐2HG	CCDC86	TCTA	RYBP
HIST2H2BE	HIST2H2BC	DUSP1	CSF2	RSRC2	EPC1	GEM	DEFB1	NSDHL
CXCL3	HIST1H2BE	RIPK4	TNNC1	HIST1H2BN	HSPB8	SBDS	PRMT5	ZNF821
HIST1H2BO	IFIT2	CKLF‐CMTM1	LOC105373383	TAF3	FAS	LATS2	HIST1H2AE	MIR31HG
HIST1H2AK	CYTOR	S100A3	NFKBIE	URB1‐AS1	SLC25A22	AKAP17A	RELB	PITX2
HIST2H2AA4	TUBB2A	LIN28A	HIST1H4J	MYL9	JOSD2	SFPQ	SCAF4	SLC25A25
HIST2H2AA3	TFAP2A‐AS2	RND3	HIST1H2AC	DNAJC2	LSMEM1	NR2F2	MUC2	
KRTAP3‐1	GADD45G	LOC101928841	GPR87	LYAR	AURKAPS1	MYL2	NOV	
CXCL1	GPR3	NR4A1	H19	IGFBP6	ATAD3B	CLTB	AKR1B10	
ARRDC3	SNAI2	HES1	C6orf226	SESN1	NXF1	RBM38	DAGLB	
SMIM11A	TP53I3	RPL21	DUSP14	LINC00431	IFNA22P	MRPL23	C1orf116	
ZC3H12A	SCARNA28	LOC654342	PNP	PCNA‐AS1	PURPL	RPL23AP82	CMYA5	
CTGF	CD14	VGF	PPM1D	RNR1	FAM53C	ZNF622	CLU	
CXCL2	SNHG9	SERPINB2	TRMT6	SNRPA1	CHN1	TRIML2	PARTICL	
SLMO2‐ATP5E	TUBA1A	ARL14	NFYC‐AS1	TYW1B	ATOX1	DKK4	WDR63	
HIST1H2BJ	GADD45A	NGFR	GPRC5D	THUMPD3‐AS1	PER1	TIGAR	PDX1	
U2AF1L5	NR4A3	ALPP	PTAFR	COTL1	HIST1H3H	LMO7	ELOA	
ZNF296	TCTE3	HIST3H2A	MDM2	DDN	PMAIP1	DANCR	KTI12	
HIST1H2BK	DDX39A	STAM‐AS1	HMOX1	FUBP1	PCF11	CRISPLD2	LTV1	
HIST1H2BC	UPK3B	SERPINB5	SNHG15	LGALS7B	IER3	PSPC1	LINC02273	
CYR61	FAM46B	GAL	PDLIM2	SNRNP25	TAF7L	RPS19BP1	SNF8	
SCAANT1	HIST1H3D	FGFBP1	MIR22HG	ASH1L‐AS1	IFIT1	PAK1IP1	FLNC	
KRTAP2‐3	EGR2	MED26	CCZ1P‐OR7E38P	LINC02004	CDKN3	OSR2	CCNB1	
HIST1H1C	SERF2‐C15ORF63	LINC02486	MAFF	MT4	FBLL1	KBTBD8	PSMD2	
CXCL8	SIK1	HIST1H2AI	AEN	CBWD5	PER2	LIMA1	IL32	
WFDC2	RRAD	ZNF703	KLF11	FBXW7	NME1	LY6D	FAM133DP	
BHLHE41	BTG2	TRIAP1	HIST1H4K	HBEGF	OSGIN1	LOC101928131	HIST1H1E	
PLK2	TUBA4A	KLF10	ZNF674‐AS1	SLC16A14	SLC30A1	POLE3	IFNA1	
SNAI1	SSSCA1‐AS1	IER2	ALYREF	PGF	KIF20A	CAPN10‐AS1	SNHG19	
SNORA94	THBS1	CD83	FOSB	PHLDA3	DGUOK‐AS1	VIM	GALR2	
FAM25A	RASL11A	GAS6‐AS2	MSX2	TUBB3	LSM3	CDC25A	ZBTB2	
SERPINE1	SCARNA9L	ODC1	YTHDF3‐AS1	TNKS2‐AS1	HIST2H3D	SDC1	HOXC5	
DHRS2	GALNT5	DLX2	ALPPL2	ARRDC4	SERPINI1	APOD	TUBA1B	
RGS16	TUBB2B	CCL20	CYP1A1	LOC100506082	ABHD11‐AS1	LINC01004	CXCR4	
FOS	S100A7	KLK5	MYH16	UBE2S	FOSL1	CAVIN1	LINC01481	
SNORA58	JUN	EID3	TAGLN3	HMGCS1	CITED1	LAMA3	ATP6V0C	
CPA4	SFN	BHLHE40	AMOTL2	EPHA2	PIGW	FTH1P3	SPP1	
ATF3	LOC100506358	PRDM1	FAM83C‐AS1	ANKRD33B	S100A2	RPL17‐C18orf32	RPS14P3	
NR1D1	LOC644656	SNHG1	NME1‐NME2	FGF8	ZBTB49	WNT9A	CRSP8P	
TNFAIP3	PDE6G	NUAK2	LGALS7	HIST1H2BM	LOC105370941	MTA2	ING1	
GAST	EGR1	MOBP	KRT15	LOC100270804	LOC102724428	NCBP2‐AS2	TUBA1C	
HBA1	HIST1H4E	VHLL	EIF3C	TUBB4A	LOC105369340	PINX1	SRPX2	
NFKBIA	EGR3	LOC101927765	PDRG1	ALOXE3	EFNB1	NAP1L5	IDI1	
HIST1H2BD	ADM	SRRT	APOE	TSPYL2	CSF1	H2AFJ	TM4SF19	
AQP3	NOCT	ARC	PLK1	MGARP	FABP5	ZNF654	HES2	
HIST2H2BF	TUBB6	HIST1H2AM	S100A10	EHD1	SUSD2	CDKN2D	IDH1‐AS1	
HIST3H2BB	ACTA1	MAP2K3	RBBP6	PTTG1	LOC100507412	FOXD4L3	TGS1	
CCL26	S100A5	ZNF79	SNHG20	LOC100272217	CEBPD	HIST1H2AH	PSMA7	
RFPL3S	KHDC1L	CA2	SRSF2	HIST1H2AD	POP1	COX7B	TFRC	
LINC00115	BIRC3	TRIM29	TNFRSF10C	BBC3	TSGA10	BUD31	C19orf73	
CDKN1A	MAGEA2B	HBA2	LINC01186	CLP1	AURKA	SCML1	DKK1	
JUNB	ACHE	CSRNP1	DCAF4L1	C16orf91	TRMT61A	LOC730202		

CBT vs Control downregulated genes						
SNORA4	CA9	ERICH2	ESRP1	STOX1	PRTN3	MTHFD2
SNORA81	PLA2G4B	ULBP1	CSPG4	RHBDD1	PRR36	MCOLN3
SNORA23	AMT	KIFC2	C6orf48	NUCKS1	WARS	SYCE1L
BCYRN1	MOCOS	IDUA	PGGHG	CHKB	SMIM14	HEXDC
RMRP	PHGDH	HSF4	LOC729737	L3MBTL1	DHTKD1	SRGAP2C
SNORA53	RHBDL1	MACROD1	GSTM4	EBF4	ATF5	ATG16L2
SCARNA5	SLPI	MIR210HG	FER1L4	RPS6KA2	CCNL2	IFI27L1
RPPH1	LOC155060	CCDC18‐AS1	FGF19	KLK7	GOLT1A	LINC01730
FAM129A	LHPP	FHL1	LINC01063	NQO1	TNFRSF6B	SGK2
LOC692247	CDK5RAP3	CCDC57	FAM229A	DDX12P	FARP1	BACE1
PABPC1L	SLFN5	CPT1B	RHPN1	GP1BA	ST6GALNAC2	PITPNA‐AS1
SCARNA10	THSD4	LINC02482	OLFM2	LINC01564	HOXC4	YJEFN3
RNA5‐8SN4	KCNQ1	THBS3	ALPK1	ASAP3	SPX	ARPIN
PCK2	EPOR	GIPR	SLC27A1	PLOD2	MOB3A	LOC171391
ABCC3	ANGPTL4	CDRT4	LRMDA	CEBPG	NPIPB15	MLLT6
SNORA73B	TRIM74	ABCG1	ALDH3B1	NEIL1	IFI35	NOXA1
KLHDC7B	PAQR6	DAPK2	DOC2A	CIRBP	EXD3	CYTH2
RBCK1	MLXIPL	MST1R	TBC1D3J	GLG1	BRCA1	CERS6‐AS1
GTPBP2	GSTM2	ANKRD37	SREBF1	GNG7	C19orf66	SEL1L
LOC109864269	HIST1H4I	PSAT1	TRPV6	MYO1D	TMEM198B	MISP3
LCN2	R3HDM2	QPCTL	NIT1	SNTB1	FAM131C	CACNA1H
LOC102725254	C1RL	MTMR9LP	AARS	IGFLR1	NSUN5P1	IFITM1
TRIB3	MUSTN1	LTBP4	CFAP74	SLC25A35	TSSK3	PDE2A
CCPG1	RPS10P7	OR2A9P	OR2A4	SLC23A3	ATP6AP1L	SLX1B
SPRR2D	KIF21B	OR2A7	DTD1	SLC45A1	JDP2	UCN
HIST2H2AC	SLC22A31	MKNK2	PET100	PRSS53	SCARNA9	GSEC
HIST1H4A	GALNT12	SLC7A1	TOX2	LHB	TMEM268	CACNA2D2
CHAC1	FIBCD1	DHRS11	LINC01133	GSTO2	LONP1	GFPT1
XLOC_007697	TLCD2	ARHGEF2	P4HA1	TSPAN18	ENKD1	C1QTNF6
NBR2	H6PD	SERPINA5	CAMK1D	FOXC1	GAA	
LAMP3	PTPRCAP	LOC642846	CPQ	RNF144A	PAN2	
IL20RB	FUT3	CALB2	GNAZ	PKD1	PTP4A3	
PTPDC1	NRBP2	LINC01775	GOLGA8A	PTK6	FLJ23867	
MAPK15	BRICD5	FBXO36	NTSR1	HLCS	SYNPO	
PIP5KL1	SYTL1	C15orf65	NREP	SGSM2	NQO2	
SCARNA6	RNA18SN3	RAD9A	DMGDH	TMEM200B	KIAA0895L	
LEMD1	CRYL1	ERRFI1	ACCS	IL11RA	MAFA‐AS1	
LURAP1L	SLC6A9	BNIP3L	MMP25‐AS1	HDAC6	LRRC75B	
OR2A20P	MLPH	BCKDHA	WDR27	SKAP1	ZNF395	
HKDC1	ENO2	IZUMO4	PLAUR	EML2	SLC9A3‐AS1	
GAS5	KLK10	ARSG	ANO9	TRIM66	CCDC146	
SLC7A11	ATP2C2	TRIOBP	NUP210	SLC2A3	SPRY4‐AS1	
MEGF6	RGL3	NUDT8	CBLC	HIGD1B	HIST1H4B	
PRPH	GRAMD1B	ANKRD19P	SLC16A5	VEGFA	GRB7	
LOC103021295	PYGB	KCTD15	SEC31B	LINC01503	OCEL1	
ASNS	CLGN	TMEM254	FAM86B1	LARP6	GOLGA8B	
TMC4	FBXO48	LRRC56	ECI1	TNFRSF25	LOC102724404	
AKNA	PAQR4	EMID1	LOC100505585	TNRC6C‐AS1	ARHGEF19	
SLC1A4	ADAMTS10	MYO15B	NRSN2‐AS1	KLF2	ROBO3	
SLC22A18	TMEM91	ITGA3	ALDOC	R3HDM4	JMJD7	
KCNG1	NRP1	ASIC1	TAZ	DDIT4	ZFAS1	
	CTBS	KLF9	PHYKPL	HIST2H2AB	SLC43A1	

Gene Ontology (GO) analysis on molecular function term enrichment revealed that CBT treatment led to the upregulation of genes involved in a variety of binding events, such as protein binding, protein dimerization, DNA binding and organic cyclic compound binding, etc. (Fig. 4B). The abnormal binding events indicate the disruption of normal dynamics of the microtube lattice inside the cells. Indeed, GO analysis revealed that multiple TUBB and TUBA family genes were upregulated in CBT‐treated HCT116 cells (Fig. 4C). This might be a result of the inhibition of the disassembly of the microtube by CBT forcing the cells to complementarily express microtube assembly‐related genes. A function chord diagram further revealed that Tubb3, Tubb6, Tubb2a, Tubb4a and Tubb2b are linked to the microtubule‐based process, the response to an external stimulus, and the mitotic cell cycle process (Fig. 4D), suggesting a disruptive role of CBT on these processes.

### Cabazitaxel inhibits colorectal cancer cell growth via activating the p53 signaling pathway

**Fig. 5:**
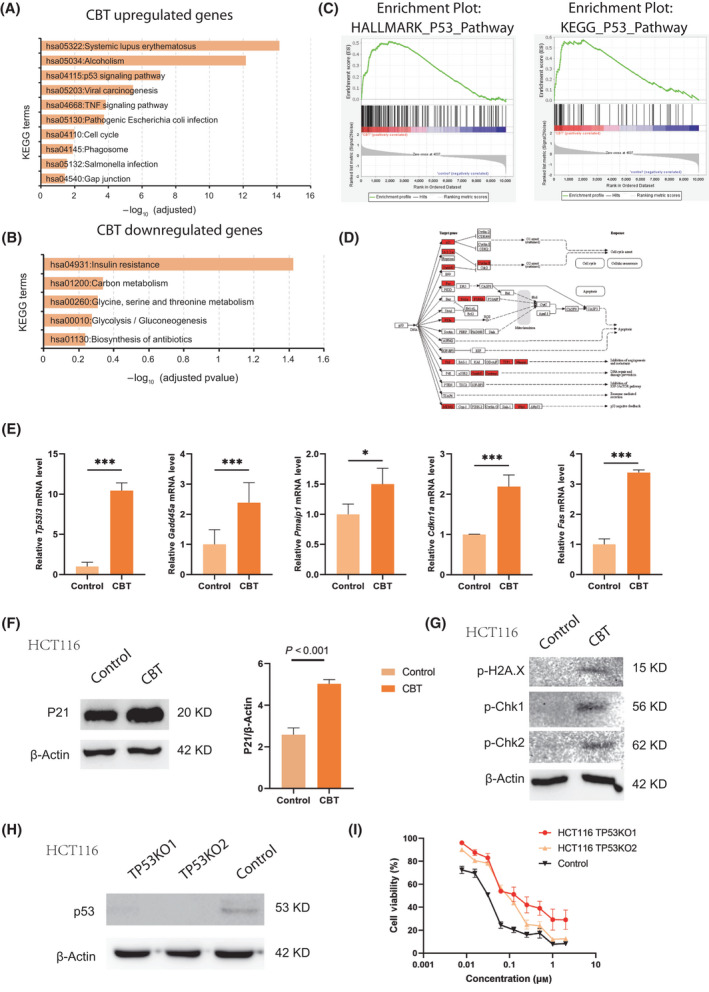
Cabazitaxel induces upregulation of the p53 pathway. (A) KEGG pathway analysis of the upregulated gene enriched biological pathways of CBT‐treated HCT 116 cells compared to control HCT116 cells. (B) KEGG pathway analysis of the downregulated gene enriched biological pathways of CBT‐treated HCT 116 cells compared to control HCT116 cells. (C) GSEA diagram showing the positive correlation of p53 pathway genes and upregulated genes in CBT‐treated cells compared to control HCT 116 cells. (D) Pathview rendered p53 downstream pathway with integration of RNA‐seq data of control and CBT‐treated HCT 116 cells. Red indicates the upregulated genes after CBT treatment. (E) Real‐time PCR results showing the relative mRNA levels of Tp53i3, Gadd5a, Pmaip1, Cdkn1 and Fas in control and CBT‐treated HCT116 cells. Three biological experiments were performed for each assay. Student's *t*‐test was used for statistical analysis. ***P < 0.001. (F) Western blotting and densitometric analysis of p21 protein expression in control and CBT‐treated HCT116 cells. β‐actin was used as an internal control in the experiment. The data are shown as the mean ± SD (*n* = 3), Student's *t*‐test was used for statistical analysis. (G) Representative western blot image showing the expression of p‐H2A.X, p‐Chk1 and p‐Chk2 in control and CBT‐treated HCT116 cells. β‐actin was used as an internal control in the experiment. (H) Representative western blot image showing the expression of p53 in TP53KO1, TP53KO2 and control HCT116 cells. β‐actin was used as an internal control in the experiment. (I) *In vitro* cytotoxicity of TP53 KO cells and control HCT116 cells. The data are the mean ± SD (*n* = 6). The cell viability rate was obtained by normalizing the MTT assay output of CBT‐treated cells with the control dimethylsulfoxide treated cells.

In addition to a number of Tubb family genes being upregulated in CBT‐treated cells, KEGG pathway analysis revealed that CBT treatment‐induced genes were enriched in the well‐known antitumor p53 signaling pathway (Fig. 5A). Meanwhile, CBT treatment indicated that downregulated genes were related to multiple metabolism processes, such as carbon metabolism and glycine, serine and threonine metabolism, as well as glycolysis (Fig. 5B). Furthermore, GSEA revealed a positive correlation between p53 pathway genes and CBT upregulated genes in HCT116 cells, indicating that CBT indeed enhances the expression of p53 pathway genes (Fig. 5C). Rending the genes to the p53 pathway clearly showed that multiple cell cycle arrest‐related genes, such as p21, 14‐3‐3‐δ and Gaff45, were increased in CBT‐treated cells (Fig. 5D). p53 downstream genes Fas, Noxa, PUMA and PIGs, which induce apoptosis, and PAI, TSP1 and Maspin, which inhibit angiogenesis and metastasis, were also upregulated in CBT‐treated cells (Fig. 5D). Furthermore real‐time PCR assays also confirmed that the mRNA levels of p53 downstream genes such as Tp53i3, Gadd5a, Pmaip1, Cdkn1a and Fas were significantly higher in CBT‐treated HCT 116 cells than in control cells (Fig. 5E). In addition, the expression of p53 major downstream protein p21(Waf1/CiP1) that links DNA damage to cell cycle arrest was enhanced in CBT‐treated HCT116 cells compared to the control cells (Fig. 5F). Meanwhile, CBT treatment led to obvious DNA damage, as manifested by the expression of p‐H2A.X, p‐Chk1 and p‐Chk2 (Fig. 5G).

To examine whether activation of p53 pathway plays a key role for CBT efficacy, we used the CRISPR/Cas9 system to knock out TP53, a p53 encoding gene, in HCT116 cells by two different gRNAs and generated TP53 KO1 cells and TP53 KO2 cells. Western blotting revealed that p53 was completely depleted in the TP53 KO cells (Fig. 5H). The MTT assay revealed that the IC_50_ values of CBT to TP53 KO1 cells and TP53 KO2 cells were 0.175 and 0.096 μm, respectively, which were approximately at least 3‐fold higher than the IC_50_ of CBT in HCT116 cells. The enhanced resistance to CBT of TP53 KO cells indicates that the inhibitory effect of CBT to HCT116 cells relies on the TP53 pathway (Fig. 5I). All of these results substantiate our conclusion that CBT inhibits HCT116 cells mainly by activating the p53 pathway.

## Discussion

As a result of the resistance of colorectal cancer to current drug therapies, there is an urgent need to develop new antitumor drugs. In the present study, we found that FDA‐approved drug CBT exhibits potent antitumor efficacy to colorectal cancer. CBT is a microtubule inhibitor [28] that has been reported to bypass some cancer resistance mechanism toward chemotherapeutic agents and shows good efficacy to metastatic prostate cancer, breast cancer and ovarian cancer [4, 29, 30]. In the present study, we demonstrated that CBT also has potent antitumor function with respect to colorectal cancer.

Tubulins are the primary targets of CBT. Tubulin is the basic block of microtubes that contributes to the cytoskeleton and cell mobile elements. Hence, polymerization and depolymerization of tubulin are essential in mitosis, intracellular transport and cell movement, etc. CBT binds to tubulin and promotes microtube assembly and inhibits its disassembly. Hence, CBT seriously interferes with the recycling of tubulin and the normal dynamics of microtube networks in cells that are required for biological processes. We observed significant upregulation of Tub family gene expression, which manifests as the compensative expression of these genes by cells in response to the microtube assembly‐related units after CBT treatment. Consistent with this, we observed a series of microtube inhibition‐related cell biology abnormalities, such as cell cycle arrest, cell proliferation, and migration inhibition and apoptosis. In the end, we found that CBT efficiently inhibits the growth of HCT116 xenograft tumor. Unlike inhibition of androgen receptor and heat shock proteins in prostate cancer cells or targeting the phosphoinositide 3‐kinase/Akt/mechanistic target of rapamycin pathway in lung adenocarcinoma cells [5], CBT enhances the antitumor pathway‐p53 signaling pathway in colorectal cancer cells. p53 and its downstream genes are well characterized with resect to inducing apoptosis and senescence of cancer cells and inhibiting tumor growth and angiogenesis in cancers [31]. The p53 signaling pathway is frequently dysregulated in colorectal cancer. Approximately 40–50% of sporadic colorectal cancer harbor a p53 mutation [32]. Reactivation or restoration of the p53 pathway downstream effectors can efficiently improve the prognosis of colorectal cancer. In line with the apoptotic phenotype triggered by CBT, we found that CBT treatment leads to activation of multiple p53 downstream target genes, such as apoptosis activating genes including Gadd45a [33], Tp53Ii3 [34] and Pmaip1 [35]. The marker for DNA damage, p‐HA2.X was also elevated in CBT‐treated HCT116 cells. p‐H2A.X not only recruits proteins involved in DNA repair, but also correlates with apoptosis. As a consequence of apoptosis, DNAs are fragmented and trigger the phosphorylation of H2A.X. Hence, p‐H2A.X levels can be used to monitor the anticancer therapy effect as well. An increase in p‐H2A.X in CBT‐treated HCT116 cells demonstrates the efficacy of CBT with respect to anti‐colorectal cancer at the molecular level. DNA damage generally activates p53 and its major downstream target p21 and leads to cell cycle arrest. An increase in p21 in CBT‐treated HCT116 cells confirms the activation of the p53‐p21 pathway. To support our conclusion, we also generated TP53^−/−^ HCT116 cells. Compared to HCT116 cells, TP53^−/−^ HCT116 cells are more resistant to CBT treatment, suggesting that CBT inhibitory effect to HCT116 cells relies on the P53 signaling pathway. Furthemore, p53 mutated HCT8 cells and DLD1 cells are more resistant to CBT treatment than HCT116 cells and LoVo cells also demonstrate the need for p53 signaling so that CBT can exert its function in colorectal cancer cells. We also noted that multiple metabolism processes of HCT116 were also disturbed by CBT. A well known characterisitic of cancer cells is that they adopt special metabolic features. The disturbance of these features would affect cancer cell survival, proliferation and migration. Detailed mechanistic studies of the effect of CBT on the metabolism of colorectal cancer are needed in the future.

In the present study, we have shown that CBT can efficiently inhibit colorectal cancer proliferation and migration. It suppresses colorectal cancer via enhancing the expression of multiple p53 downstream effector genes and promoting cell cycle arrest, apoptosis and inhibition of angiogenesis. Hence, CBT may serve as an alternative option for colorectal cancer treatment in the future.

## Conflict of interest

The authors declare no conflict of interest.

## Author contributions

PY and JP designed the study. WZ and RS performed the key experiments. RH, QL, WW and XC carried out the supportive experiments. YZ and JZ performed the bioinformatics analysis. WZ and PY analyzed the data and wrote the manuscript. All authors read and approved the final version of the manuscript submitted for publication.

## Data Availability

The RNA‐seq data that support the findings of the present study are openly available in the Sequence Read Archive (SRA) (https://www.ncbi.nlm.nih.gov/sra) under accession number PRJNA687151.
