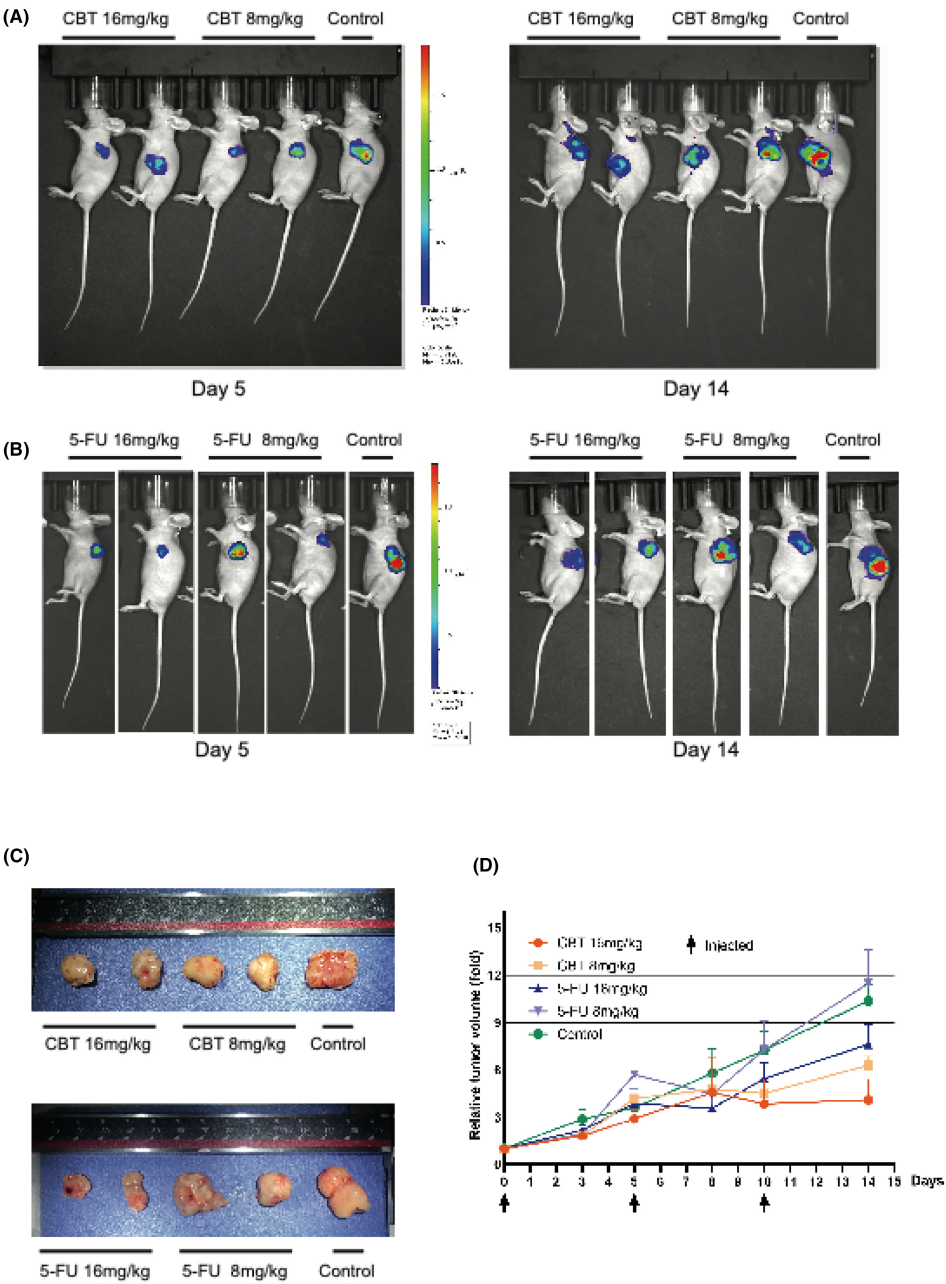# Correction to ‘Cabazitaxel suppresses colorectal cancer cell growth via enhancing the p53 antitumor pathway’

**DOI:** 10.1002/2211-5463.70256

**Published:** 2026-04-22

Wen Zhang, Ruiqian Sun, Yongjun Zhang, Rong Hu, Qian Li, Weili Wu, Xinyu Cao, Jiajian Zhou, Jianfeng Pei, Ping Yuan. Cabazitaxel suppresses colorectal cancer cell growth via enhancing the p53 antitumor pathway. *FEBS Open Bio*. 2021;11(11):3032–3050, doi: https://doi.org/10.1002/2211‐5463.13290.

A concerned reader identified an assembly error in Figure 3B of the above article. In the panel showing Day 5 bioluminescence imaging, the image assigned to the 5‐FU 16 mg/kg group was incorrectly replaced with a duplicate of the image used for the 5‐FU 8 mg/kg group. The correct Figure 3, with the proper image for the 5‐FU 16 mg/kg group at Day 5 in Figure 3B, is provided below.

This error occurred during figure assembly and does not affect the results, conclusions or integrity of the study.

We apologize for this error.